# Exploring the Motivations for Completing Advance Care Directives: A Qualitative Study of Majority/Minority Israeli People Without Dementia

**DOI:** 10.3389/fpsyt.2022.864271

**Published:** 2022-03-14

**Authors:** Perla Werner, Natalie Ulitsa, Hanan AboJabel

**Affiliations:** Department of Community Mental Health, University of Haifa, Haifa, Israel

**Keywords:** dementia, advance care directives, advance care planning, end-of-life, majority and minority groups, laypeople

## Abstract

**Background:**

Completing advance directives has been declared an essential instrument for preserving and respecting the autonomy and preferences for end-of-life care of people living with dementia. However, research deciphering the reasoning behind the decision to complete or not advance directives in the case of dementia remains limited, especially among people pertaining to different majority/minority groups.

**Objectives:**

To explore the motivations of people without dementia in Israel to complete or not to complete advance directives and to compare these motivations among the majority veteran Jewish group, the minority Jewish Former Soviet Union immigrant group, and the minority Arab group.

**Methods:**

This qualitative study used purposive sampling and focus groups with discussions elicited by a vignette. A total of 42 Israeli people without dementia participated in 6 focus groups: two with veteran Jews (*n* = 14), two with Jewish immigrants from the Former Soviet Union (*n* = 14), and two with Arabs (*n* = 14). The analysis followed recommended steps for thematic content analysis.

**Results:**

Four overarching themes were identified: (1) the meaning of dementia-related advance directives, (2) motivations for willingness to complete advance directives, (3) motivations for not being willing to complete advance directives, and (4) ethical dilemmas. Some of the themes were common to all groups, while others were informed by the groups' unique characteristics. Participants displayed a lack of knowledge and misunderstanding about advance directives, and central concepts such as autonomy and competence. Furthermore, stigmatic images of dementia and of the person with the diagnosis were associated to participants' motivations to complete advance directives.

**Conclusions:**

There is need to expand comparative research among culturally and socially similar and dissimilar groups within a country as well as between countries in order to better guide public health efforts to increase the rates of advance directives completion. Special attention should be paid to decreasing stigmatic beliefs and understanding unique cultural values and motivations.

## Introduction

The conceptualization of dementia has continuously evolved over time. From the use of a predominantly biomedical model, stressing the pathological aspects of the condition and seeing the pursuit of a cure as the first priority, the understanding of dementia is guided today by a *relational model* based on an enablement and equality approach, stressing the principles of self-determination, autonomy, human rights, and involvement in decision-making (1). Implementing advance care planning and completing advance directives are fundamental to attain the goals of this new dementia framework.

### End-of Life and Advance Care Planning

Advance care planning is the process of discussing and communicating to family members and physicians personal predilections regarding end-of-life medical care, in the event that the individuals in question lose the capacity to make medical decisions or express their own wishes. *Advance directives* are legal documents describing preferences for future care and appointing a surrogate to make health care decisions in the event of incapacity (2). Today, advance care planning and advance directives are conceptualized as a health behavior (3), and their benefits are demonstrated at the individual (e.g., reducing unnecessary pain, unhelpful or invasive procedures or unwanted hospitalizations), family (e.g., minimizing stress and burden, avoiding conflicts among family members, and providing peace of mind), physician (e.g., reducing conflicts and misunderstanding with family members, avoiding ethical dilemmas), and social levels (e.g., reducing health care costs) (4–9). Given the characteristics of dementia (10), completing advance directives has been declared an essential instrument for preserving and respecting the autonomy and preferences for end-of-life care of people with this disease (11). However, the rates of completion and use of advance directives in general and in the case of dementia in particular, are low (12).

The general public and professionals' limited knowledge and feelings of discomfort and reluctance to talk about end-of-life issues, as well as the complexities of appropriately completing, storing, and implementing the documents are among the most common barriers to the completion of advance directives in general (13–15). Predicting the time of death, determining when the person has lost the capacity to express medical treatment preferences, and corroborating whether the wishes expressed prior to the loss of decisional-capacity still reflect the person's values and legacy are among the main unique challenges confronting the implementation of advance directives in dementia (16). However, research deciphering the reasoning behind the decision to complete advance directives or not in the case of dementia remains limited (13), especially among people belonging to different cultural or religious groups. This matter is of extreme importance, especially as previous studies have shown cultural, religious, and majority/minority differences in rates of completion and attitudes toward advance directives, as well as in end-of-life values in general (17–19).

### Advance Care Planning in Israel

In Israel, there are two ways of formally undertaking advance care planning: advance directives and power of attorney. These procedures were established by Israel's Ministry of Health within the framework of the “Dying Patient Law” enacted in 2005. According to this law, patients are defined as *terminally ill* if they are suffering from an incurable disease and have a life expectancy of up to 6 months. Although attention to palliative care in general, and advance care planning in particular, has developed over recent years in Israel (20–22), >1% of the adult population has formally completed advance directives or signed a durable power of attorney (23).

Israel is a multicultural society comprising a majority Jewish group and a minority, mostly Arab, group. The Arab group is often seen as being characterized by higher levels of collectivism and familism than is the Jewish group (24), although it is undergoing rapid processes of modernization (25).

Moreover, the majority Jewish group itself is also not monolithic; indeed it is diverse in terms of culture and language, and is characterized by a variety of immigrant groups. People from the former Soviet Union (FSU) constitute the largest immigrant group (26). The majority of them immigrated to Israel in the early 90-ies of the last century and were characterized by relatively old age, a high level of education, small households size, and a strong tendency to preserve the Russian language and cultural norms and values from the country of origin (27–29).

### Study's Objective and Research Questions

The overall aim of the study was to explore the motivations of people without dementia in Israel to complete or not to complete advance directives and to compare these motivations among the majority veteran Jewish group, the minority Jewish Former Soviet Union immigrant group, and the minority Arab group.

Our specific research questions were: (1) What motivates people without dementia to endorse completing or not completing advance directives? (2) Do these motivations vary among people belonging to the majority veteran Jewish group, to the FSU immigrant Jewish group, or to the Arab minority group?

## Methods

### Study Design

We conducted a qualitative study using focus groups. This method was selected because of its suitability to obtain an in-depth understanding of participants' opinions when discussing sensitive and complex topics such as dementia-related advance care directives (30).

### Participants

The study included 42 Israeli participants. Inclusion criteria were: being at least 18 years old, not having a diagnosis of dementia, and belonging either to the majority group in Israel (veteran Jewish), or to one of the largest minority groups (Jews from the FSU, and Arabs).

### Procedure and Sampling

We conducted 6 focus groups from October 2018 to March 2019. Participants were enlisted using purposive sampling and were recruited via researchers' professional and personal connections. As suggested in the literature (30), focus groups included six to eight participants. All participants signed informed consent before the initiation of the focus groups.

### Data Collection

Focus groups were organized separately for each of the three groups. Discussions began with the researchers asking the participants to introduce themselves and talk about their personal experiences with dementia and with people with the disease. This was followed by the presentation of a vignette (see Appendix 1), portraying a person in his late sixties who decides to prepare advance care directives because of the chance of getting dementia. Immediately after asking about their general understanding of the meaning of advance directives, groups' coordinators explained to the participants the meaning of the concept. Probes were used to prompt the opinions, experiences, emotions, and thoughts provoked by the person in the vignette. Focus groups lasted on average 90 min and were conducted by researchers with experience in qualitative research. To ensure data trustworthiness, discussions were conducted in the native language of the participants until saturation was reached. Furthermore, discussions were audio-recorded and then transcribed verbatim. Detailed coding guidelines, including verbatim pseudonymized quotes, were translated into Hebrew (if necessary) and then into English.

### Data Analysis and Triangulation

The analysis included several steps for thematic content analysis (31). First, transcribed discussions were read several times. Second, the whole data set was systematically coded in accordance with the main study topic: participants' reasons for completing or not completing advance directives. We identified categories and codes through inductive coding; and discerned main themes. Coding sheets (compiled in tables) enabled quotations to be organized by themes, categories and codes. Categories and codes were discussed within the research group until a consensus was reached.

### Ethical Considerations

The study protocol was approved by the University of Haifa's Ethics Committee (Ref. Nr. 384/17; 12/11/2017).

## Results

### Participants' Characteristics

A total of 42 Israeli participants were included in the study: 14 Jewish veterans—mean age = 45.92 (SD = 17.20), 71.4% female, 14 Jewish immigrants from FSU—mean age = 72.86 (SD = 9.02), 64.3% female, 14 Arabs—mean age = 45.42 (SD = 14.25), 50% female. Participants' characteristics are presented in Table 1.

**Table 1:** Participants' characteristics.

	**Jewish** **(*n* = 14)**	**Arab** **(*n* = 14)**	**Immigrants** **from FSU** **(*n* = 14)**
	**Mean (SD)**	**Mean (SD)**	**Mean (SD)**
**Age**	45.92 (17.20)	45.42 (14.25)	72.86 (9.02)
**Number of years of education**	16.57 (2.17)	12.14 (4.58)	14.86 (2.07)
**Number of children**	2.28 (1.38)	2.57 (2.06)	1.64 (0.74)
	***n*** **(%)**	***n*** **(%)**	***n*** **(%)**
**Gender**			
Male	4 (28.57)	7 (50.0)	5 (35.7)
Female	10 (71.43)	7 (50.0)	9 (64.3)
**Marital status**			
Married	10 (71.43)	10 (71.42)	6 (42.86)
Not married	4 (28.57)	4 (28.28)	8 (57.14)
**Economic situation**			
Below average	0	5 (35.71)	1 (7.14)
Average	2 (14.29)	8 (57.15)	12 (85.72)
Above average	12 (85.71)	1 (7.14)	1 (7.14)

### Themes

We were able to identify four overarching themes: the meaning of dementia-related advance directives, motivations for the willingness to complete advance directives, motivations for not being willing to complete advance directives, and ethical dilemmas.

#### Theme 1. Understanding the Meaning of Advance Directives

In general, all participants lacked a clear understanding about the meaning of advance directives. Veteran Jewish participants did not differentiate between a last will and advance directives. “*It's a last will” (VJ, LP5, 28)*. Some of them even described advance directives as a path to euthanasia: “*These (advance directives) are clear guidelines leading to euthanasia” (VJ, LP12, 69*). A participant in the Arab group defined advance directives as “*…murder out of pity”* (*Arab, AB5, 30*). Only a few participants differentiated between euthanasia and extending life artificially, and only one participant knew that euthanasia was not legal in Israel: “*This topic is irrelevant because in Israel there is no such law (euthanasia)”* (*FSU, RU2, 62*).

#### Theme 2. Reasons for Being Willing to Complete Advance Directives

Overall, 23% of the participants expressed being interested in completing advance directives: 21% among veteran Jewish participants, 21% among participants from the FSU, and 28% among Arabs. The rest were not interested in completing ACD (and even opposed it) or did not express a clear opinion on the matter.

We identified three subthemes that served as motivations for completing advance directives among our sample:

##### Subtheme 2.1: Avoiding Suffering

Mitigating pain and suffering—especially for family members—was an important subtheme for Jews from the FSU, and for the Arab group.

“*I think that (completing advance directives) will help avoid suffering for those around him (person in the vignette), and also for himself.” (FSU, RU15, 77)*.

“*It (dementia) is a disease that causes much pain to the person and the family, so there is need to complete advance directives” (Arab, AB6, 45)*.

The motivation to complete advance directives as a means of avoiding pain and suffering was closely related to the participants' perceptions of dementia. For example, Jews from the FSU and Arabs made a clear distinction between the necessity of completing advance directives for dementia vs. for other diseases. They emphasized that in the case of dementia, because of the lack of a potential cure, completing and respecting advance directives is obligatory; while in reference to a treatable disease there is need to respect the persons' wishes, even if not conveyed in a legal document.

“*He (the person in the vignette) is at an advance stage of an incurable disease. If additionally, he suffers from another disease, there is need to respect anyway his wishes” (FSU, RU12, 69)*.

Participants from the Arab sector, however, felt that when people have a potentially curable disease, their preferences as reflected in the document, should not be respected.

“*If it is a disease that can be treated… if there is an available medication, then you can't respect the advance care directives. But if there is no cure and the disease causes great pain, then one must respect the directives” (Arab, AB6, 45)*.

##### Subtheme 2.2: Maintaining Autonomy

One of the main subthemes emerging as a motivation to complete advance directives was ensuring that the person living with dementia could retain a sense of control, of make independent choices, and have a sense of agency. This subtheme was mentioned by all of the participants, who emphasized that choices must be made by the person living with dementia as long as the person still has the ability to do so.

“*He (person in the vignette) has the full right to decide how he wants to end his life. He is cognitively intact now, so he can make a decision rationally and not as a consequence of the dementia” (VJ, LP11, 69)*.

“*He's making his own decisions (completing advance directives); and they certainly have to be respected. If he thinks this is what is good for him, then his wishes have to be fulfilled” (FSU, RU8, 78)*.

“*He is in a good cognitive situation. He can make decisions by himself. We ought to respect these decisions” (Arab, AB4, 58)*.

##### Subtheme 2.3: Honoring the Person

Although all of the participants elicited the topic of autonomy, only the Arab group mentioned completing and respecting advance directives for the sake of honoring people (mainly older people).

“*We have to honor older people. It's very important” (Arab, AB11, 61)*.

“*For us, honor is the most important matter.” (Arab, AB9, 57)*.

##### Subtheme 2.4: Stigmatic Beliefs About Dementia

All participants expressed stigmatic beliefs toward people living with dementia. They described them as “*… a body without a soul.” (Jew, LP8, 18)*; and “*a vegetable” (FSU, RU2, 62);* or “*crazy” (Arab, AB1, 33)*. Stigmatic beliefs increased participants' willingness to complete advance directives, especially as a means of reducing unnecessary anguish, or unnecessarily extending a “useless” life*: “I think it is worthless to hold onto the body, something material without a soul. There is a need to free the body… there is a need to have advance directives and respect them” (Jew, LP8, 18).”*

#### Theme 3. Reasons for Not Being Willing to Complete Advance Directives

Participants who opposed to completing or respecting advance directives felt that end-of-life decisions shouldn't be made by the people living with dementia themselves, but by others. However, who these “others” were, varied by the participant group. Jews from the former FSU considered that only the **physicians** should be able to make end-of-life decisions:

“*The person can write whatever s/he wants, but at the right moment the physicians will decide according to the situation” (FSU, RU10, 86)*.

This belief stemmed mainly from participants' attitudes toward the role of the physician as the person who has the knowledge and the responsibility to decide for their patients:

“*The physician should not listen to the patient. He (the physician) ought to make the decisions!” (FSU, RU11, 82)*.

“*Who wants to take this (making end-of-life decisions) responsibility? For this, there is a physician who has taken an oath to fight for the patient till the last second of his/her life, regardless of what the patient wants.” (FSU, RU9, 62)*.

Participants from the Arab sector indicated that only **family members and God** could make these decisions:

“*I would suggest that (the person in the vignette) not sign an advance directives. Only family members can decide. Doesn't matter what disease it is; there is a need to always act in accordance with the family's wishes.” (Arab, AB8, 22)*.

“*I wouldn't do it (complete advance directives). We believe in God's decisions” (Arab, AB11, 61)*.

#### Theme 4. Ethical Dilemmas

Regardless of their willingness to complete or not complete advance directives, participants discussed ethical problems associated with the implementation of these documents. This theme comprised three subthemes, the findings of which—similar to previous ones—varied by participant group.

##### Subtheme 4.1: Difficulties in Determining Cognitive Functioning

Participants in the veteran Jewish focus groups debated about who, how, and when is determined that people can no longer make decisions for themselves, and when advance directives should be implemented.

“*This (preparing advance directives) has to be very clear and specific; otherwise the physician will decide by himself. Who should decide if I'm incompetent? And how?” (VJ, LP10, 72)*.

“*This is a good question! Who should decide if the person is in an advance stage of dementia? And when to implement the advance directives?” (VJ, LP6, 48)*.

##### Subtheme 4.2: Change of Preferences

Participants in the veteran Jews and former FSU groups discussed the moral dilemma that occurs after having completed the advance directives, and in the context of cognitive deterioration, the people change their minds and express different end-of-life preferences than the ones documented in the advance directives.

“*It is a well-known phenomenon…. people might talk this way (i.e., don't want to continue living if they are in a severe stage of dementia), but when the time comes, there is a twist in the plot and they change their minds, and suddenly they don't think the same way anymore”. (Jew, LP10, 72)*.

“*My father always said – I want this and this (to end his life). But when you get to the finish line in life – you want to remain alive. There is no way of knowing what he really wanted at the last minutes of life”. (FSU, RU4, 70)*.

##### Subtheme 4.3: Family Members' Differing Views

Participants in the veteran Jews and Arab groups voiced concerns stemming from potential disagreements between the family members themselves, or between family members' preferences and the directives expressed by the person living with dementia.

“*We had a difficult situation with my father. We had to decide whether to agree or not on resuscitating him. The eldest son was the one who had to decide, but no one knew what he wanted. It was not clear at all” (Jew, LP6, 46)*.

“*The problem with advance directives is that some relatives want to respect them and others don't. This might cause a lot of conflicts between relatives” (Arab, AB5, 51)*.

## Discussion

Respecting how a person wants to die is a fundamental human and moral claim, even for people living and dying with dementia (32). Advance directives are the most recognized and available tools for protecting the autonomy of people living with dementia by communicating their values and medical care preferences to family members and physicians while they are still able and competent to make decisions (33). The contribution and feasibility of these legal documents might be more important today than in the past, as knowledge and research in the development and clinical use of biomarkers continues to move forward, allowing a prodromal diagnosis of dementia, and permitting people to express and communicate their preferences before or at very early stages of the disease (34). However, the completion and implementation of advance directives worldwide has been far from satisfactory (12).

In the current study, we explored the motivations to complete or not complete dementia-related advance directives, while at the same time comparing majority/minority groups based on cultural, religious and immigration differences. Overall, our findings showed that some of the themes emerging from the data were common to all groups, while others were informed by the groups' unique characteristics.

### Understanding Dementia-Related Advance Directives

Knowledge and perceptions are core elements of most social-cognitive models of health behavior (35). Thus, it is not surprising that the meaning of advance directives and dementia were extensively discussed in the groups.

Similar to previous studies examining perspectives regarding advances care planning in general (36, 37) and advance directives in particular (38), all participants lacked a clear understanding of the concepts. They tended to conflate advance directives with people's last will or testament, and those in the veteran Jewish and Arab groups even equated them with euthanasia. This finding represents a major problem for two reasons. First, a recent integrative review reported that many studies that have been conducted in different countries and cultures found that lack of awareness, clarity, and knowledge serve as the main barriers for the initiation and implementation of advance care planning (13). Second, confusing advance directives with euthanasia might dramatically reduce the willingness to completing these documents, especially among veteran Jewish and Arab participants as Judaism and Islam (monotheistic religions) perceive life as a gift from God and strongly oppose both passive and active euthanasia (39). This was less evident among Jews from the FSU, who despite being Jews are less attached to religion. Thus, if the aim is to promote the completion and use of advance directives, there is a need to find ways of increasing knowledge on this topic.

### Reasons for Completing or Being Willing to Complete Advance Directives

*Avoiding or relieving pain* was one of the main motivations driving participants to complete or being willing to complete advance directives. This finding is not surprising given that, as suggested by the self-determination theory (40), avoiding or relieving pain is a basic human need across the life-course and as the end-of-life approaches, in particular. However, whereas in other diseases the wish to avoid suffering is conceptualized mainly as an intrinsic motivation (41), our participants referred to preventing pain not as a reward for themselves but for those surrounding them. Indeed, McAfee and colleges (42), in a study assessing advance care planning behaviors among a racially diverse sample of 386 American adults aged 40 and above, found that participants diagnosed with a life-threatening illness or those having witnessed a patient with such a disease, behaved on the basis of extrinsic motivations. Our results show that this pattern can be generalized to people with no exposure to a life-threatening disease, as many of our participants were in fact not exposed to a life-threatening disease.

The willingness to complete advance directives was associated with the participants' views about dementia and about its effect on the diagnosed person. Negative perceptions of dementia as an incurable disease, and of the person with the disease as “soulless”, increased participants' respect for the person's end-of-life preferences and seemed to bolster for them, the importance of advance directives. These results may suggest that people without dementia in Israel, regardless of their religion, culture, and migrant status adhere to the biomedical model of dementia which, as stated, focuses on the disease process (1).

The importance of completing advance directives to *maintain the person's autonomy* was a prominent theme among all participants, regardless of their background. This finding is not surprising given that preserving people's dignity and self-determination, and ensuring that their medical preferences and values are respected, is a central principle of advance directives in general (13), and for persons with dementia in particular (43). However, participants raised three ethical problems associated with the completion and implementation of advance directives as a tool for securing autonomy and self-agency of people living with dementia at the end-of-life. Their first worry related to the difficulties in determining when people have lost the capacity to make decisions for themselves, and regarding who has the knowledge and authority to make this determination. The second dilemma referred to the problem in determining whether the preferences expressed by people in their advance directives while considered competent, would remain unchanged when cognitive capacity to make decisions is lost, and advance directives need to be implemented. These concerns reflect the ongoing ethical and moral debate between bio-ethical scholars about the meaning of *competence* and decision-making capacity in the case of dementia, about whether the identity of a person living with dementia is preserved or not (i.e., the *personal identity argument*), and about whether the aim of advance directives is to maintain the person's *precedent or current autonomy* (44–46). It should be noted that these dilemmas were expressed only by veteran Jewish participants, most probably because of their higher level of education.

The last problem discussed by our participants related to potential conflicts that can emerge between family members when interpreting and implementing advance directives. Family disagreements are common among relatives when making end-of-life decisions for a person living with dementia (47). Moreover, research has demonstrated that family struggles regarding what is the best quality of life and the best quality of dying for their relatives are associated with increased caregiver burden and distress (48). Such struggles do not, of course, reduce the suffering of family members; a motivation that was mentioned by our participants as one of the main reasons for completing dementia-related advance directives. This finding suggests that our participants understand that enacting legal documents such as advance directives is not enough to fulfill the reasons for doing so, rather additional steps are needed, as we will discuss further.

### Reasons for Not Completing or Not Being Willing to Complete Advance Directives

This theme emerged only among participants belonging to the two minority groups in the study (the FSU and Arabs groups), who expressed the idea that end-of-life decisions should not be made by the individuals with dementia themselves. Nevertheless, whereas immigrants from the FSU felt that only physicians should make these decisions, Arab participants shared their belief that only family members and God were authorized to make end-of-life decisions. This difference most probably stems from cultural differences among the groups. On the one hand it can be suggested that immigrants from the FSU are driven by an authoritarian medical model, where discussions with physicians or shared-decision making are totally absent and only physicians make decisions (49). On the other hand, Israeli Arab participants opposed the completion of advance directives because of their strong familism and religious values (50, 51). These results support efforts to expand knowledge about the influence of cultural factors, values and beliefs on attitudes regarding advance care planning (19, 42).

### Limitations of the Study

Our study has a few limitations that need to be acknowledged. First, participants were recruited using convenience sampling, and most of the focus groups were conducted in the northern area of Israel. Thus, as with many qualitative studies, the generalizability of our findings is limited. Most noteworthy are the age differences between the former Soviet immigrants (in their 70s) and the two native groups (in their mid-40s), as well as the gender distribution between the groups—i.e., the higher proportion of female participants in the Jewish groups compared to Arab participants. Thus, we can't rule out the possibility that beyond cultural differences, these socio-demographic characteristics affected participants' perceptions of the end-of-life options. Future studies should use more representative samples to disentangle these effects.

Second, some of the participants had a personal, albeit not close acquaintance with the researchers, potentially introducing social desirability bias. That said, we did not see any direct evidence of such a bias in the discussions, during which participants spoke freely and critically. Third, while exploring the motivations of a diverse sample of people without dementia is one of the main strengths of our study, it should be noted that the Arabs group was composed exclusively of Muslims, and did not include Christians or Druze participants. Although Muslims constitute 84% of the Arab sector in Israel, future studies should include other denominations as well. Finally, our findings have to be interpreted in light of the unique legal, moral and religious characteristics of Israel. Despite these limitations, our study provides important information for understanding the reasoning behind the decision to complete or not complete advance directives.

### Conclusion and Implications

In light of the continued rise in the number of people living and dying with dementia worldwide (52), and the new models stressing the importance of respecting the autonomy and self-determination of people living with dementia, this qualitative study explored motivations of Israeli people without dementia to complete or not to complete advance directives, while focusing on similarities and differences between majority/minority groups. As demonstrated in other studies, all our participants displayed a lack of knowledge and great misunderstanding about advance care directives. However, more importantly they also exhibited a superficial understanding of central concepts such as autonomy and competence, which were mostly perceived by them as dichotomies rather than as relative, time-specific and task-specific concepts (16, 53).

This study suggests a number of future research and practical directions. First, our findings clearly illustrate the need to expand comparative research among culturally and socially similar and dissimilar groups within a country as well as between countries in order to better guide public health efforts to increase the rates of advance directives completion. Second, the motivations for completing advance directives should be examined also among groups with direct familiarity with dementia, such as people in the early stages of the disease and their family members. Third, future studies should thoroughly examine the development and effectiveness of interventions aimed at encouraging the completion of advance directives. Special attention should be paid to efforts in decreasing stigmatic views of dementia, as well as taking into consideration the unique values underlying the motivations for completing advance directives, especially among majority and minority groups.

## Data Availability Statement

The raw data supporting the conclusions of this article will be made available by the authors, without undue reservation.

## Ethics Statement

The studies involving human participants were reviewed and approved by University of Haifa. The patients/participants provided their written informed consent to participate in this study.

## Author Contributions

PW designed the study, supervised data collection and data analysis, and wrote the first draft of the paper. NU collected the data, conducted data analysis, and assisted in the writing of the manuscript. HA collected the data and assisted in the writing of the manuscript. All authors contributed to the article and approved the submitted version.

## Funding

This study was funded by a grant from the German–Israeli Foundation for Scientific Research and Development (GIF) (Grant Number G-1413- 119.4/2017).

## Conflict of Interest

The authors declare that the research was conducted in the absence of any commercial or financial relationships that could be construed as a potential conflict of interest.

## Publisher's Note

All claims expressed in this article are solely those of the authors and do not necessarily represent those of their affiliated organizations, or those of the publisher, the editors and the reviewers. Any product that may be evaluated in this article, or claim that may be made by its manufacturer, is not guaranteed or endorsed by the publisher.

## Appendix 1: Case Vignette

*Imagine the following scenario: A 67-years old teacher decides to complete advance care directives because of the concern of being diagnosed with dementia in the future. In the advance directives document, he specifies that he will object any end-of-life medical treatment if being in an advanced stage of dementia*.

## References

[1] Werner P Van Gorp B Vermeulen P Simonsen P. From History to Intervention: A Socio-Cultural Analysis of Dementia Stigma. Cambridge: Cambridge University Press. (2021).

[2] Kermel-Schiffman I Werner P. Beliefs of Israeli family caregivers of people with dementia toward advance care planning. J Soc Work End Life Palliat Care. (2020) 16: 250–65. 10.1080/15524256.2020.1745729 32251608

[3] Carney MT Williams M Zhang M Kozikowski A Dolgin J Kahn A. Impact of a community health conversation upon advance care planning attitudes and preparation intentions. Gerontol Geriatr Educ. (2021) 42:82–95. 10.1080/02701960.2020.1739670 32223366

[4] Baker A Leak P Ritchie L Lee A Fielding S. Anticipatory care planning and integration: a primary care pilot study aimed at reducing unplanned hospitalization. Br J Gen Pract. (2012) 62:113–20. 10.3399/bjgp12X625175 22520788 PMC3268490

[5] Brinkman-Stoppelenburg A Rietjens JA Van der Heide A. The effects of advance care planning on end-of-life care: a systematic review. Palliat Med. (2014) 28: 1000–25. 10.1177/0269216314526272 24651708

[6] Carlson EA. Three books on improving organizational functions. Orthop Nurs. (2017) 36:67–70. 10.1097/NOR.0000000000000314 28107305

[7] Detering KM Hancock AD Reade MC Silvester W. The impact of advance care planning on end-of-life care in elderly patients: randomized controlled trial. BMJ. (2010) 340:c1345. 10.1136/bmj.c1345 20332506 PMC2844949

[8] Schubart JR Reading JM Penrod J Stewart RR Sampath R Lehmann LS. Family caregivers' characterization of conversations following an ACP event. Am J Hosp Palliat Care. (2018) 35:1161–7. 10.1177/1049909118760302 30071784 PMC6092939

[9] Spivey C Brown TL Courtney MR. Using behavioral economics to promote advance directives for end-of-life care: a national study on message framing. Health Psychol Behav Med. (2020) 8:501–25. 10.1080/21642850.2020.1823227 34040883 PMC8114389

[10] Alzheimer's Association. Alzheimer's disease facts and figures. Alzheimers Dement. (2020) 16:391–460. 10.1002/alz.12068 32157811

[11] Alzheimer's Association. Alzheimer's disease facts and figures. Alzheimers Dement. (2014) 10:e47–92. 10.1016/j.jalz.2014.02.001 24818261

[12] Piers R Albers G Gilissen J De Lepeleire J Steyaert J Van Mechelen W. Advance care planning in dementia: recommendations for healthcare professionals. BMC Palliat Care. (2018) 17:1–17. 10.1186/s12904-018-0332-2 29933758 PMC6014017

[13] Hemsley B Meredith J Bryant L Wilson NJ Higgins I Georgiou A. An integrative review of stakeholder views on advance care directives (ACD): barriers and facilitators to initiation, documentation, storage, and implementation. Patient Educ Couns. (2019) 102:1067–79. 10.1016/j.pec.2019.01.007 30799141

[14] Werner P Schiffman IK. Attitudes and completion of advance care planning: Assessing the contribution of health beliefs about Alzheimer's disease among Israeli laypersons. Palliat Support Care. (2019) 17:655–61. 10.1017/S1478951519000336 31131787

[15] Perin M Ghirotto L De Panfilis L. ‘Too late or too soon': the ethics of advance care planning in dementia setting. Bioethics. (2021) 35:178–86. 10.1111/bioe.12814 33283327

[16] Hart D. 203-decisional capacity and advance care planning in older people who are incarcerated. Int Psychogeriatr. (2021) 33:8–8. 10.1017/S104161022100137X

[17] Clark MA Person SD Gosline A Gawande AA Block SD. Racial and ethnic differences in advance care planning: results of a statewide population-based survey. J Palliat Med. (2018) 21: 1078–85. 10.1089/jpm.2017.0374 29658817

[18] Pereira-Salgado A Mader P O'Callaghan C Boyd L. A website supporting sensitive religious and cultural advance care planning (ACPTalk): formative and summative evaluation. JMIR Res Protoc. (2018) 7:e8572. 10.2196/resprot.8572 29661749 PMC5928329

[19] Sinclair C Smith J Toussaint Y Auret K. Discussing dying in the diaspora: attitudes towards advance care planning among first generation Dutch and Italian migrants in rural Australia. Soc Sci Med. (2014) 101:86–93. 10.1016/j.socscimed.2013.11.032 24560228

[20] Bar-Sela G Mitnik I Zalman D Flechter E Sheinman-Yuffe H Vulfsons S. Medical students' attitudes towards participating in a palliative medicine course: a new specialty in Israel. Palliat Support Care. (2018) 16:528–33. 10.1017/S1478951517000943 29198227

[21] Bentur N Sternberg S. Implementation of advance care planning in Israel: a convergence of top-down and bottom-up processes. Gerontologist. (2019) 59:420–5. 10.1093/geront/gnx157 29045631

[22] Feder SL Collett D Haron Y Conley S Meron T Cherny N. How skilled do Israeli nurses perceive themselves to be in providing palliative care? Results of a national survey. Int J Palliat Nurs. (2018) 24:56–63. 10.12968/ijpn.2018.24.2.56 29469647

[23] Shvartzman P Reuven Y Halperin M Menahem S. Advance directives—the Israeli experience. J Pain Symptom Manage. (2015) 49:1097–101. 10.1016/j.jpainsymman.2014.12.009 25637243

[24] Abu-Asbah K Raian-Garaa N Abu Nasra M. Psychological treatment in Arab society in Israel: Between tradition and modernity. Hevra v'Revaha (Soc Welfare): Soc Work Quart. (2014) 34: 101-121. (In Hebrew)

[25] A'li N Da'as R. Arab women in Israeli politics: aspirations for fundamental equality or preservation of gender inequality. Cult Relig Stud. (2016) 4:67–86. 10.17265/2328-2177/2016.02.001

[26] Israeli Central Bureau of Statistics. The Statistical Abstract of Israel. Jerusalem: CBS Publishing (2017).

[27] Elias N. Russian-speaking immigrants and their media: still together? Isr Aff. (2011) 17:72–88. 10.1080/13537121.2011.522071

[28] Remennick L. Professional identities in transit: factors shaping immigrant labor market success. Int Migr. (2013) 51:152–68. 10.1111/j.1468-2435.2011.00733.x

[29] Kushnirovich N. Wage gap paradox: the case of immigrants from the FSU in Israel. Int Migr. (2018) 56:243–59. 10.1111/imig.12490

[30] Stewart DW Shamdasani PN Rook DW. Group dynamics and focus group research. in Introduction: Focus Group History, Theory, and Practice. (2015) 3:3–19. 10.4135/9781412991841.d3

[31] Braun V Clarke V. Using thematic analysis in psychology. Qual Res Psychol. (2006) 3:77–101. 10.1191/1478088706qp063oa

[32] Dildy KC Largent EA. Directing the end of life in dementia. In: Dubljević V Bottenberg F editors. Living with Dementia: Neuroethical Issues and International Perspectives. Cham: Springer (2021). p. 71–89.

[33] Porteri C. Advance directives as a tool to respect patients' values and preferences: discussion on the case of Alzheimer's disease. BMC Med Ethics. (2018) 19:1–8. 10.1186/s12910-018-0249-6 29458429 PMC5819243

[34] Scharre DW. Preclinical, prodromal, and dementia stages of Alzheimer's disease. Pract Neurol. (2019) 37–47.31164314

[35] Gellert P Tille F. What do we know so far? The role of health knowledge within theories of health literacy. Europ Health Psychol. (2015) 17:266–74. Available online at: https://www.researchgate.net/publication/299543089

[36] Fried TR Paiva AL Redding CA Iannone L O'Leary JR Zenoni M. Effect of the STAMP (Sharing and Talking About My Preferences) intervention on completing multiple advance care planning activities in ambulatory care: a cluster randomized controlled trial. Ann Intern Med. (2021) 174:1519–27. 10.7326/M21-1007 34461035 PMC8711627

[37] Martina D Geerse OP Lin CP Kristanti MS Bramer WM Mori M. Asian patients' perspectives on advance care planning: A mixed-method systematic review and conceptual framework. Palliat Med. (2021) 35:1776–92. 10.1177/02692163211042530 34488509 PMC8637390

[38] Laranjeira C Dixe M Gueifão L Caetano L Passadouro R Querido A. Awareness and attitudes towards advance care directives (ACDs): an online survey of portuguese adults. Healthcare. (2021) 9:648. 10.3390/healthcare9060648 34072558 PMC8227883

[39] Yildirim JG. Knowledge, opinions and behaviors of senior nursing students in Turkey regarding euthanasia and factors in Islam affecting these. J Relig Health. (2020) 59:399–415. 10.1007/s10943-019-00954-z 31768823

[40] Ryan RM Deci EL. Self-determination theory and the facilitation of intrinsic motivation, social development, and well-being. Am Psychol. (2000) 55:68. 10.1037/0003-066X.55.1.68 11392867

[41] Richards N. Assisted suicide as a remedy for suffering? The end-of-life preferences of British “suicide tourists.” *Med Anthropol*. (2017) 36:348–62. 10.1080/01459740.2016.1255610 27845576

[42] McAfee CA Jordan TR Sheu JJ Dake JA Kopp Miller BA. Predicting racial and ethnic disparities in advance care planning using the integrated behavioral model. Omega. (2019) 78:369–89. 10.1177/0030222817691286 28142319

[43] Donnelly S Begley E O'Brien M. How are people with dementia involved in care-planning and decision-making? An Irish social work perspective. Dementia. (2019) 18:2985–3003. 10.1177/1471301218763180 29544346

[44] Jongsma K. Losing rather than choosing: a defense of advance directives in the context of dementia. Am J Bioeth. (2020) 20:90–2. 10.1080/15265161.2020.1781957 32757919

[45] Jongsma KR Kars MC van Delden JJ. Dementia and advance directives: some empirical and normative concerns. J Med Ethics. (2019) 45:92–4. 10.1136/medethics-2018-104951 29907577

[46] Widdershoven G Janssens R Voskes Y. Beyond precedent autonomy and current preferences: a narrative perspective on advance directives in dementia care. Am J Bioeth. (2020) 20:104–6. 10.1080/15265161.2020.1781969 32757936

[47] Cresp SJ Lee SF Moss C. Substitute decision makers' experiences of making decisions at end of life for older persons with dementia: a systematic review and qualitative meta-synthesis. Dementia. (2010) 19:1532–59. 10.1177/1471301218802127 30253658

[48] Moore KJ Davis S Gola A Harrington J Kupeli N Vickerstaff V. Experiences of end of life amongst family careers of people with advance dementia: longitudinal cohort study with mixed methods. BMC Geriatr. (2017) 17:1–13. 10.1186/s12877-017-0523-3 28673257 PMC5496359

[49] Telen MJ. Teaching evidence-based medicine in the former Soviet Union: lessons learned. Trans Am Clin Climatol Assoc. (2014) 125:88. 25125721 PMC4112671

[50] Ayalon L. Family relations and elder care among Arabs in the North of Israel. Res Aging. (2018) 40:839–58. 10.1177/0164027517749612 29303044

[51] Ayalon L Karkabi K Bleichman I Fleischmann S Goldfracht M. Between modern and traditional values: informal mental health help-seeking attitudes according to Israeli Arab women, primary care patients and their providers. Int J Soc Psychiatry. (2015) 61:386–93. 10.1177/0020764014549082 25205778

[52] Barbarino P Lynch C Watchman K Dabas L Arthurton L. From plan to impact IV: progress towards targets of the WHO Global action plan on dementia. London: Alzheimer's Disease International (2021).

[53] Werner P Schicktanz S. Competence and cognitive deterioration: are we paying enough attention to ethical issues? In: Schweda M Pfaller L Brauer K Adloff F Schicktanz S editors. Planning Later Life: Bioethics and Public Health in Ageing Societies. New York, NY: Routledge (2017). p. 89–103.

